# Measuring User Experience, Usability and Interactivity of a Personalized Mobile Augmented Reality Training System

**DOI:** 10.3390/s21113888

**Published:** 2021-06-04

**Authors:** Christos Papakostas, Christos Troussas, Akrivi Krouska, Cleo Sgouropoulou

**Affiliations:** Department of Informatics and Computer Engineering, University of West Attica, 12243 Athens, Greece; cpapakostas@uniwa.gr (C.P.); akrouska@uniwa.gr (A.K.); csgouro@uniwa.gr (C.S.)

**Keywords:** augmented reality, firefighting training, technology acceptance model (TAM), factor analysis, regression analysis, usability, user experience

## Abstract

Innovative technology has been an important part of firefighting, as it advances firefighters’ safety and effectiveness. Prior research has examined the implementation of training systems using augmented reality (AR) in other domains, such as welding, aviation, army, and mathematics, offering significant pedagogical affordances. Nevertheless, firefighting training systems using AR are still an under-researched area. The increasing penetration of AR for training is the driving force behind this study, and the scope is to analyze the main aspects affecting the acceptance of AR by firefighters. The current research uses a technology acceptance model, extended by the external constructs of perceived interactivity and personalization, to consider both the system and individual level. The proposed model was evaluated by a sample of 200 users, and the results show that both the external variables of perceived interactivity and perceived personalization are prerequisite factors in extending the TAM model. The findings reveal that the usability is the strongest predictor of firefighters’ behavioral intentions to use the AR system, followed by the ease of use with smaller, yet meaningful, direct and indirect effects on firefighters’ intentions. The identified acceptance factors help AR developers enhance the firefighters’ experience in training operations.

## 1. Introduction

Augmented reality (AR) is defined as a system that has the following properties: (a) combines real and virtual objects, (b) is interactive in real-time, and (c) is registered in three dimensions [1]. Feiner et al. [2] described the term AR as an enhancement of the real world by a virtual world, which provides valuable information, such as descriptions of important features or instructions for performing physical tasks. Milgram et al. [3] discussed AR displays in a general sense, and defined AR as augmenting natural feedback to the operator with simulated cues. Drascic and Milgram [4] examined the meaning of the term AR, and proposed AR displays as being the image of a primarily real environment, which is augmented with computer-generated imagery.

AR has been explored in all levels of education [5,6], providing great advantages such as student motivation and learning effectiveness [7,8,9,10]. AR is on the verge of transforming the human–computer relationship. AR contributes to interactivity and facilitates co-creation [11]; thus, AR has the potential to create training environments and scenarios that are cost-effective, safe and personalized. This represents a good fit with the training carried out for firefighters. The immersive nature of the training using mixed reality offers a unique realistic quality, which is not generally present in traditional education in the classroom, yet retains considerable cost advantages over large-scale real-life exercises and is gaining increasing acceptance [12].

The first potential of integrating AR in firefighting training is the cost effectiveness. Indeed, current firefighting training in the real environment requires important costs in infrastructures, such as a large training area, propane-fed firefighting props, fire extinguishers, ventilation, and ladders. The training facility has to deal with complex and varied scenarios, and their replication needs vehicles, dumpsters, pipes, containers, and even apartment floorplans. AR can handle all these infrastructures, providing considerable savings in consumables; thus, the preparation time for each training scenario is significantly reduced.

Secondly, the use of AR leverages the trainees’ experiences to a wide variety of training scenarios. The firefighting area varies widely depending on the category and, as such, there is aerial support of wild firefighting [13], urban firefighting [14], and ship firefighting [15]. The aforementioned scenarios can be covered in an AR training process, giving the trainees the opportunity to repeat the training session and advance their skills in firefighting.

Apart from the cost effectiveness and the variety of scenarios offered, the AR adoption has no environmental charge, no uncontrolled fires, toxic gas emission, or heat burst. AR does not entirely supplement traditional training exercises; however, it offers the possibility to train in high-risk scenarios, without actually encountering any physical risk of the trainees’ health. Despite the reported side effects experienced by the head-mounted display (HMD) [16], most users face no symptoms, while only a few experience minimal discomfort in training environments.

For all the aforementioned advantages, using AR to train firefighters is a novel training approach that will improve the training of qualified firefighters. Some preliminary studies have already been conducted in the field of firefighting training [15,17,18,19]; however, the conditions under which firefighters may accept the technology of the developed systems still remain unclear. In this context, there is a lot of room for improvement in this area. The current research aims to evaluate the use of AR technology in training firefighters, which is an area that has not been thoroughly investigated. At the same time, the evaluation is carried out using a novel model, which was adequately extended to emphasize the human element in relation to the system (human–computer interaction) and context (personalization).

We assessed the acceptance of AR technology for firefighting training, by proposing an acceptance model specific for a “Naval Fire Fighting Training & Education System” (NAFTES) (https://etraining.naftes.eu/, accessed on 5 April 2021) AR application. NAFTES is freely available through the Google Play store. The suggested model includes the basic TAM variables, namely, usefulness, ease of use, and intention to use, extended by two more constructs, namely, perceived interactivity and personalization, which are addressed to evaluate the human–AR system interaction and the contextual affordances to support the training of the ship crew and officers. NAFTES was developed for two different variants, suitable for mobile (smartphones, tablets) and wearable devices (HoloLens), aiming to help trainees achieve their learning goals. The trainees can either select the mobile variant to use their personal devices and interact in the augmented ship space, or combine the AR glasses and see-through lenses. Furthermore, the system holds personalization in its incorporated mechanisms, since it provides tailored information to the users, to help towards fulfilling their learning needs. During the training phases, the system visualizes objects, information, and videos that are available to the users, according to their location, activity and actions, and delivers the corresponding assessment units to them. Regarding the level of expertise, the system offers further help to inexperienced users and supports them during their interaction with it. In the case of a diagnosed difficulty in using the application, the system proposes the use of the AR Walker application to familiarize the participants with the equipment. Moreover, it provides two different simulation scenarios for novice and advanced users, in order to match their different levels of proficiency. As such, the system provides a high degree of adaptability to trainees who have the ability to select the learning path, based on their interest, existing knowledge or the demands of their role.

NAFTES assumes the following different facets: (a) introductory training (IT); (b) theoretical (classroom) training (THT); and (c) hands-on training (HOT). IT is based on an advanced learning management system (LMS), which allows the trainees to be introduced in the fire training fundamentals. THT is supplemented by multimedia material (videos, animations, slides), which is to be integrated in the LMS, and retrieved and presented during classroom hours. Finally, HOT is an advanced portable system, which will be carried by trainees during their presence in the simulated engineering room. The system is in the form of human–AR applications, which will overlay warnings or explanatory text to the trainees’ viewport. Such applications will be executed in optical head-mounted displays (Google glasses), which will retrieve material from the LMS. While creating 3D environments is not new, developers have traditionally had to use 2D interfaces to build 3D experiences. Using AR, the full 3D space is used, which is unprecedented, and defines many of the imminent changes in human–computer interaction (HCI). Another important feature of such devices is their indoor localization capability. The NAFTES platform is complemented by a post-training assessment module, which is appended in the IT–THT–HOT workflow.

The AR firefighting training (AR-FFT) application provides two fire simulation scenarios, namely, an electric fire and a cooking oil fire. The participants have already been trained in theory, and are familiar with the protocol they have to follow in each of the aforementioned cases. The AR application helps them to evaluate their knowledge in a simulation environment. The users navigate in fire-prone areas in a ship by using QR codes. The trainees have to follow printed marks, placed in bright spots near the corresponding equipment they describe, which reveal useful instructions. The personnel are familiarized with the firefighting equipment and processes. The application also provides information material and questions on the corresponding theory, aimed at self-evaluation.

**Figure 1:**
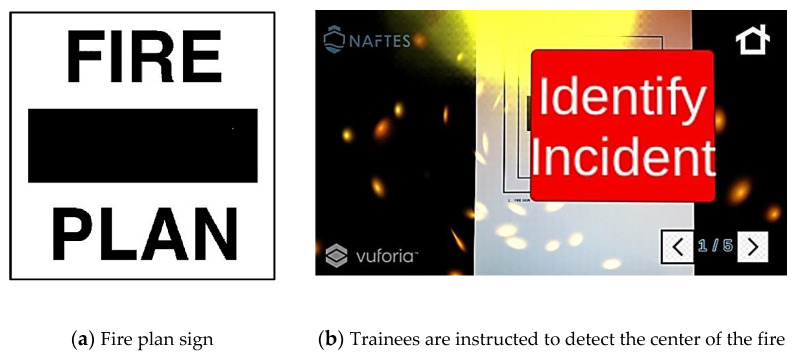
First screen of a scenario of fire on an electrical circuit. (**a**) Printed target sign, and (**b**) the corresponding instructions that appear in the firefighters’ view.

For example, the instructor chooses to simulate the electric fire scenario, and places five signs (fire plan, telephone, power relay, extinguisher, and exit door) in specific positions on the ship deck. The trainees start the training simulation, searching for the center of the fire (Figure 1), a common start point for both the electric and cooking oil fire scenarios.

**Figure 2:**
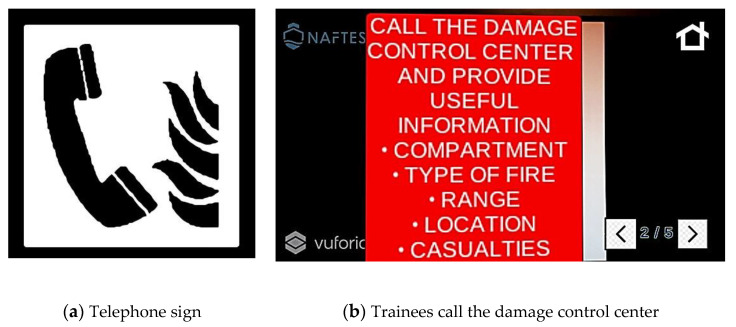
Second screen of a scenario of fire on an electrical circuit. (**a**) Printed target sign, and (**b**) the corresponding instructions that appear in the firefighters’ view.

Afterwards, further instructions to follow are displayed, and the trainees have to find the next correct check point and follow the new instructions that appear. According to the protocol, on incident identification, the personnel must find the nearest telephone to report the occasion to the damage control center and provide useful information (Figure 2).

**Figure 3:**
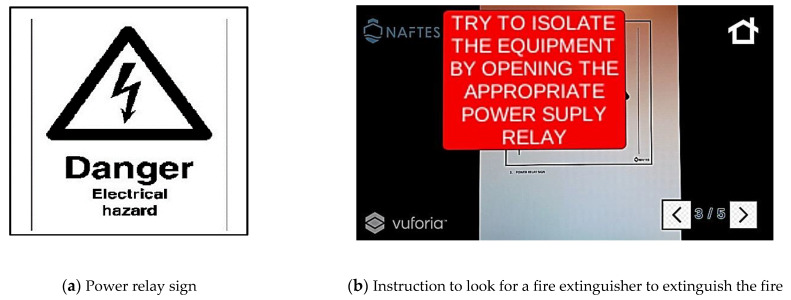
Third screen of a scenario of fire on an electrical circuit. (**a**) Printed target sign, and (**b**) the corresponding instructions that appear in the firefighters’ view.

**Figure 4:**
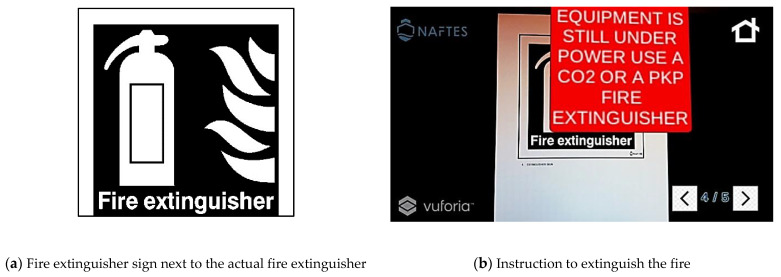
Fourth screen of a scenario of fire on an electrical circuit. (**a**) Printed target sign, and (**b**) the corresponding instructions that appear in the firefighters’ view.

The next steps of the safety protocol are displayed in Figure 3 and Figure 4. The personnel are advised to make effort to isolate the equipment, as they know that the case of an electric fire precedes the switching off of the power switch before looking for a fire extinguisher to fight the fire.

**Figure 5:**
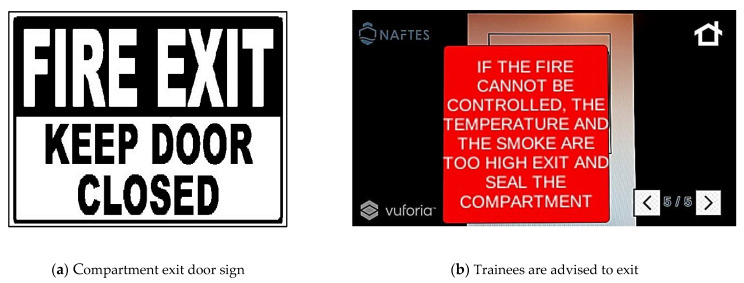
Fifth screen of a scenario of fire on an electrical circuit. (**a**) Printed target sign, and (**b**) the corresponding instructions that appear in the firefighters’ view.

Finally, the scenario suggests that in the case of no meaningful results regarding the previous actions, all the personnel should exit the dangerous area and seal the compartment (Figure 5).

When completing all the check points correctly, the trainees have successfully accomplished the particular protocol.

## 2. Literature Review

Davis [20] proposed a technology acceptance model (TAM), which consisted of two independent variables, namely, perceived usefulness (PU) and perceived ease of use (PEOU), as the main predictors of the user’s attitude towards using the system (AT), which influences the behavioral intention to use the system (BI). The undermentioned studies have examined the acceptance of AR applications in educational settings, adding particular extensions and aspects to the core TAM.

The related literature review is structured into the analysis of six different studies on AR acceptance in educational settings, and the possible extensions of their models. Yusoff et al. [21] evaluated the use of a mixed reality prototype, named the mixed reality regenerative concept, in the training of biomedical science students. The study was based on a modified TAM, incorporating the external variables of perceived innovativeness and perceived enjoyment. The findings showed positive correlations between the constructs; however, the results needed further advanced regression analysis to better evaluate the proposed model.

Wojciechowski and Cellary [22] presented a 3D image-based AR learning environment called ARIES. The evaluation of the system was based on TAM, enhanced with the constructs of perceived enjoyment and interface style. The study on the attitude of the learners toward ARIES showed great potential for education; however, more experimental studies should be carried out in order to determine the achievement of the learning goals.

Wang et al. [23] conducted a case study of forty-one aviation students in order to evaluate their acceptance of AR instructions and the future utilization of AR maintenance manuals in aviation training. The authors were based on the simplified four-construct TAM, excluding the actual system use and external variables. The study was one of the first ones to incorporate TAM in aviation training, and the findings showed that the students accepted the integration of AR in their educational settings.

Mao et al. [24] evaluated officers’ acceptance of an AR-based military decision-making process (ARB-MDMP) training system, which provided realistic troop patterns, terrain environment, and battlefield intelligence. The research model was based on TAM, and two external variables were added, namely, interface style and perceived situation awareness. The findings were encouraging and showed that the ARB-MDMP system had significant effectiveness for the officers during their courses.

Ibili et al. [25] developed a mobile AR application to teach geometry, and examined the mathematics teachers’ level of acceptance of the tutoring system. The authors extended the TAM model by including the external variables of anxiety, social norms, and satisfaction. The results supported nine out of a total of fourteen hypotheses, providing important implications for future AR tutoring applications.

Papakostas et al. [26] examined the integration of AR simulation in vocational education and training (VET), specifically in the field of industrial manufacturing. The authors were based on a modified TAM, and extended it by two external variables in order to evaluate the use of a marker-based AR application, named Soldamatic, in welding training. The findings showed that both of the external variables, namely, perceived enjoyment and system quality, were strong predictors of the proposed model, and evaluated pedagogical affordance and technological innovation at the same time.

Although there has been a lot of research on the adoption of AR for training, few studies have concentrated on the context of firefighting training [27,28,29], which mostly examined VR-based training systems. This research details the user experience of augmented reality in firefighting training, and provides a deeper understanding of the behavioral purpose to use augmented reality in marine training. Special reference is made to the factors related to the human sensory experience, such as interactivity and personalization.

One of the main criteria of effectively managing a naval fire emergency is good preparedness. AR is a promising technology that assists training and improves the abilities that are needed to deal with risky and unexpected circumstances. So far, little research has been made to investigate the acceptance of such a technology by firefighters.

Contrarily, there is still much to be done to mitigate the shortcomings of AR technology in this area. Without sufficient user studies and through the use of traditional training methods, there is little real-world evidence on the actual effectiveness of AR training. The case of technology acceptance among firefighters needs to be addressed.

Consistent with the previously stated goals, the main objective of this study is to present a modified TAM model, evaluating the user experience of AR in firefighting training. The existing literature explains the acceptance of AR technology in fields such as aviation, engineering, and military, however it does not consider the specific usage constraints in the domain of firefighting. From this, it can be deduced that there is much room for future investigation in this area. To fill the gap in the literature, we propose a detailed, domain-specific acceptance model, in which both interactivity and cognitive affordances were investigated, and thus their influence on user acceptance was measured. The related research questions that form the hypotheses of the proposed model are as follows:

- Is AR acceptable by firefighters as part of their training to develop their skills? (RQ1);
- How do the technical aspects of using AR affect trainees’ experiences of using it? (RQ2);
- What effect does the AR application’s content have on the trainees’ user experience? (RQ3).

We evaluated the AR firefighting application by expanding a TAM model, according to the research statement. Furthermore, the use of augmented reality in naval training, especially in the field of firefighting, has been shown to have a high potential for making training sessions more cost-effective, safer, and scenario-independent.

## 3. Evaluation Procedure

### 3.1. Research Model and Hypotheses

**Figure 6:**
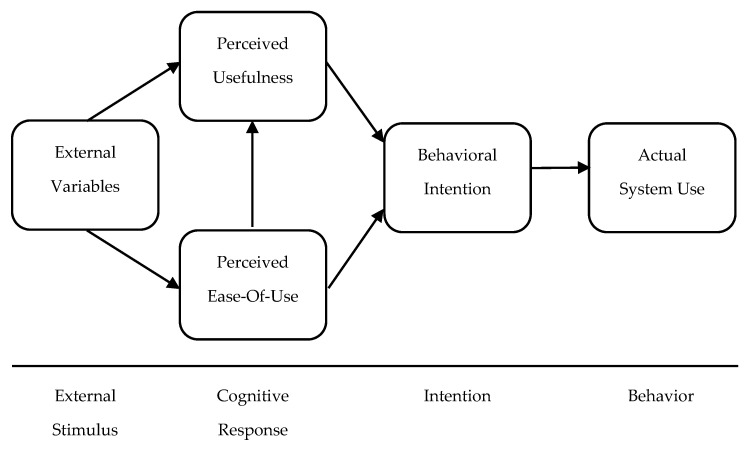
Structure of the TAM model. Reprinted with permission from ref. [31]. Copyright 1996 Elsevier.

Davis [20] proposed a model for studying the acceptance of information systems, based on the theory of reasoned action (TRA) of Fishbein and Ajzen [30]. TAM actually suggests that perceived ease of use and usefulness are the primary drivers of the acceptance of a new technology. PU is defined as the degree to which the user believes that using a particular system would enhance his/her job performance, while PEOU is defined as the degree to which a user believes that using a particular system would be free of effort. Davis and Venkatesh [31] formed the final version of TAM, suggesting that there is a causal link between external variables and user acceptance in a workplace. Behavioral intention (BI) to use a system is directly influenced by PE and PEOU, and indirectly by the external variables (Figure 6).

In this study, the final version of the model TAM, consisting of the variables of perceived usefulness, perceived ease of use, and behavioral intention, is proposed and found to be useful in predicting the use of AR for firefighting, and the following hypotheses are proposed:

**Hypothesis** **1** **(H1).** *PEOU of the AR firefighting application positively affects its PU.*

**Hypothesis** **2** **(H2).** *PEOU of the AR firefighting application positively affects its BI.*

**Hypothesis** **3** **(H3).** *PU of the AR firefighting application positively affects its BI.*

The belief of a system user may be influenced by external factors, referred to as external variables [32]. In addition to the TAM model, two external variables are integrated into the extended TAM model, with an effect on PU and PEOU.

Lee and Lee [33] provided empirical evidence supporting perceived interactivity (PI) as a prerequisite factor for extending the core TAM model, in order to understand usage behavior. McMillan and Hwang [34] measured interactivity based on three elements, namely, the direction of communication, user control, and time. The direction of communication is focused on the way that computers facilitate human interaction, emphasizing two-way communication. The user control examines the way humans control computers, while some HCI studies focus on human perception, and others on computer design [35]. The third element of interactivity is time, which evaluates the user’s ability to navigate in the application quickly and easily. An interactive system provides the users the benefits of working in their own time and choosing their preferred navigation paths [36].

In the context of the current research, PI results in firefighters’ positive beliefs about the usefulness and ease of use of the AR application, which in turn influences their behavioral intentions in the naval firefighting training. We advanced the TAM model by employing the interactivity of the crew and the officers, as a prerequisite variable, and therefore, the following hypotheses are composed:

**Hypothesis** **4** **(H4).** *PI of the AR firefighting application positively affects its PU.*

**Hypothesis** **5** **(H5).** *PI of the AR firefighting application positively affects its PEOU.*

Furthermore, personalization has been found to be a major component of many information technology-based systems [37]. Training through an AR environment can personalize learning needs in order to promote the quality of the training. AR is ideal for dangerous tasks, such as firefighting, allowing the firefighters to use dynamic learning, giving them a sense of control as they can repeat the training stages as many times as they need, learning at their own pace. Individualized, adaptive systems, facilitated by technologies such as AR, can represent the revolutionary capacity for lifelong learning, which can be incorporated into the future of training systems [38]. Hubert et al. [39] pointed out that the perceived value of personalization plays a significant role in the IT system’s acceptance.

In the context of the current research, the perceived value for personalization (PP) might play an important role with regard to the firefighters’ positive attitude towards the naval firefighting training AR application. We advanced the TAM model by employing the personalization as the second prerequisite variable of the proposed TAM model, and the hypotheses included in the model are as follows:

**Hypothesis** **6** **(H6).** *PP of the AR firefighting application positively affects its PU;*

**Hypothesis** **7** **(H7).** *PP of the AR firefighting application positively affects its PEOU.*

**Figure 7:**
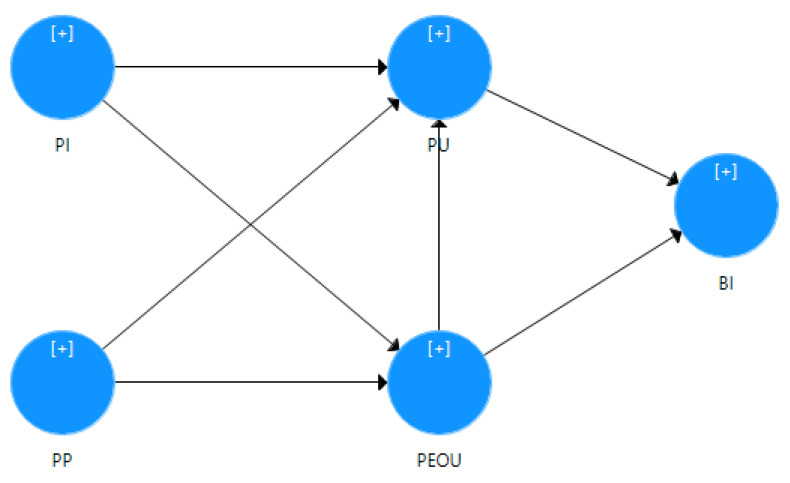
Research model flow.

The flow of the research model is shown in Figure 7.

### 3.2. Method

#### 3.2.1. Research Population

This study tested the proposed TAM model at the training of ship crews in firefighting operations. The participants were 200 volunteer firefighters, first responders, advisers working in the crisis management sector, and teachers from vocational education providers, all of whom had the opportunity to test, firsthand, the AR application of NAFTES. The AR application has been developed for both mobile devices and HoloLens glasses. The participants have been demonstrated both technologies; however, for the sake of the experiment, they used the mobile AR training system running in their own mobile devices. The reason for this decision is the cost of each equipment (i.e., mobile devices and HoloLens glasses), as HoloLens is not yet considered an economically viable approach. Their previous experience regarding the use of AR technology was just for entertainment purposes through gaming. The volunteers were aged between 18 and 60 years old. The measurements of gender and age were derived from a randomly selected sample and do not have an impact on our research findings. The demographics analysis is shown in Table 1.

#### 3.2.2. Research Instruments

The study was carried out for a period of six months, in which the participants had the chance to interact with, and validate the capabilities of, AR. The users’ feedback was evaluated in order to reveal the AR’s strengths in real applications. Data were collected via a questionnaire, which was delivered to the participants. The participants were asked to evaluate all five constructs, namely, PI, PP, PU, PEOU, and BI, by rating their training experience using a 7-point Likert scale, ranging from (1) strongly disagree to (7) strongly agree. The variables of the proposed model are assessed using indicators (Table 2), adopted from validated questionnaires [40,41,42,43] from previous related studies, and adapted according to the purpose of our study.

### 3.3. Data Analysis

The structural equation modeling (SEM) approach is used to analyze the collected data. The partial least squares (PLS) algorithm was used to build the model for the regression analysis of the variables. By estimating the individual item loadings on their respective variables, and then estimating the causal relationships among the variables, PLS allows for an optimal assessment of the measurement and structural models at the same time [46].

PLS is a strong candidate for data analysis because of the exploratory nature of our sample, where the primary goal is to understand the variance of the dependent variables induced by the independent variables, rather than the goodness of fit between the model and data [41].

In this analysis, PLS is used to test seven hypotheses and assess the relationships between the variables. SmartPLS v.3.3.3, a software tool for PLS-SEM developed by Ringle et al. [47], was used to conduct the data analysis.

## 4. Experimental Results and Discussion

### 4.1. Model Validation

In the current study, we used a reflective measurement model, which assumes that the measures represent the indication of an underlying construct [48]. We initially assessed the quality of the measurement model by using the criteria of internal consistency, convergent validity, and discriminant validity. The satisfactory results of the first phase led us use the structural model in order to test our hypotheses.

#### 4.1.1. Measurement Model

The descriptive statistics of the constructs’ indicators of the proposed model present the mean values (M), the standard deviation (SD), the excess kurtosis and skewness (Table 3). The indicators’ values provide justification for our decision, as they range from −1 to +1 for kurtosis and skewness, which are within the acceptable limits [49,50].

After the descriptive statistics analysis, we assessed the measurement model’s reliability, by measuring the indicator reliability and the internal consistency (Table 4). The indicator reliability is done by a factor analysis, which checks all the factor loadings of each indicator, and accepts the values greater than 0.700 [51]. The internal consistency can be measured either by Cronbach’s alpha (CA) or composite reliability (CR). CA is sensitive to the number of the indicators and considers each indicator to be equally reliable, while CR considers the different factor loadings of the indicators, measuring the true value of a construct’s reliability [48]. Both of the measurements, CA and CR, show a well-estimated reliability in the case where their corresponding values are greater than 0.700 [52,53]. Furthermore, Table 4 presents the assessment of the convergent validity of the constructs, made through the average variance extracted (AVE) values of each construct, which are accepted when they are greater than 0.500 [54].

Moreover, the discriminant validity assessment has the goal to ensure that a reflective construct has the strongest relationships with its own indicators. In order to examine the discriminant validity of the constructs, we firstly calculated the square root of the AVE in each variable, comparing its value to the other variables’ correlation values [55]. The model’s discriminant validity (Table 5) is valid when the AVE square root for each variable is greater than its correlations coefficients with the other factors. Secondly, Henseler et al. [56] proposed an alternative approach, based on the multitrait–multimethod matrix, to assess discriminant validity, namely, the heterotrait–monotrait ratio of correlations (HTMT). The HTMT results are shown in Table 6; when the HTMT value is below 0.900, discriminant validity has been established between two reflective constructs. 

Regarding the results shown in Table 4, we found that the factor loadings’ values ranged from 0.833 to 0.992, all of them above the 0.700 threshold, ensuring indicators’ reliability. With regards to the internal consistency reliability, the CA and CR values (Table 4) ranged from 0.902 to 0.981 and from 0.940 to 0.986, respectively, which are both greater than the threshold limit of 0.700. Therefore, the measurement model’s reliability is confirmed. As far as convergent validity is concerned, the AVE values in Table 4 were above the recommended value of 0.500, ranging from 0.839 to 0.959. Therefore, the convergent validity of the constructs is confirmed.

Lastly, the discriminant validity was measured in two parts. Based on the results shown in Table 5 of the Fornell and Larcker [55] criterion, it is evident that the diagonal values are greater than the other correlation values. Based on the results shown in Table 6 of the Henseler et al. [56] criterion, the HTMT values ranged from 0.809 to 0.882, less than 0.950, providing support for the existence of discriminant validity.

#### 4.1.2. Structural Model

After the assessment of the measurement model, we used the PLS algorithm to evaluate the path coefficients, and to test the hypotheses along with their significance levels. We ran bootstrapping estimation with the 2000 resampling method, and obtained the t-statistics corresponding to each path of our model.

**Figure 8:**
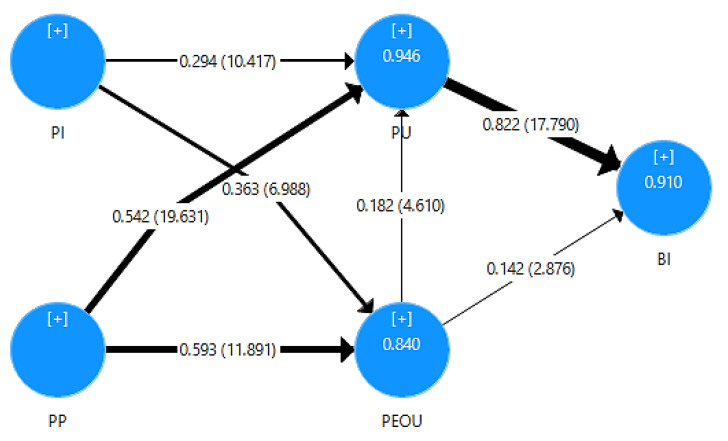
Structural model’s results.

We initially evaluated the coefficient of determination (R2). The R2 values can describe the level of predictive accuracy, as high R2 values are indicative of the quality of the model. The coefficient value of the paths of the inner model are assessed in order to measure the level of significance and show how strong the effect of a variable is on another variable. The path coefficient values range from −1 to +1, and show significance when its value is greater than 0.200 [57]. Figure 8 shows the inner model’s path coefficients and the coefficients of determination.

We used a two-tailored t-test, with a significance level of 1%. The critical t-statistics value is 2.580 for a significance level of 1% (Wong, 2013). A summary of the results from the structural model’s hypotheses testing is shown in Table 7. The following findings show that all seven hypotheses (H1 to H7) are supported: H1 (β = 0.182, *p* < 0.01), H2 (β = 0.142, *p* < 0.01), H3 (β = 0.822, *p* < 0.01), H4 (β = 0.294, *p* < 0.01), H5 (β = 0.363, *p* < 0.01), H6 (β = 0.542, *p* < 0.01), and H7 (β = 0.593, *p* < 0.01).

Two more items are indicative of the quality of the proposed model, namely, the relevance or predictive validity (Q^2^) [58,59], and the effect size (f^2^) [60]. Q^2^ is applicable only to reflective endogenous constructs [61], as BI, PEOU, and PU are in the proposed model, and its values range from negative values to greater than zero (high prediction), or ideally to one (maximum value reflecting reality). We calculated the Q^2^ values (Table 8) based on the blindfolding procedure, which is actually a technique that omits data for a given block of indicators and then predicts the omitted part based on the calculated parameters. The rule of thumb indicates that a Q^2^ greater than 0.500 is regarded as a predictive model [62].

Finally, we calculated the Cohen’s f^2^ effect size (Table 9), which is a very informative standardized measure, allowing the evaluation of local effect size, i.e., one variable’s effect size within the context of a multivariate regression model [63]. The effect sizes are interpreted as follows [60]: f^2^ ≥ 0.020, f^2^ ≥ 0.150, and f^2^ ≥ 0.350, represent small, medium, and large effect sizes, respectively.

The findings of Table 8 present Q^2^ values much higher than the cut-off limit of 0.500, indicating a highly predictive model. More specifically, BI’s Q^2^ value was calculated as 0.867, and PEOU and PU were calculated as 0.790 and 0.855, respectively. Moreover, the 91% of variance in the behavioral intention to use the AR firefighting application is defined from the usefulness and the ease to use AR technology. The 94.6% of variance in the perceived usefulness is defined from the ease of use, the interactivity and the personalization, while the 84% of variance of the perceived ease of use is defined by perceived interactivity and perceived personalization.

Regarding the f^2^ measurements in Table 9, large effect sizes are represented from PU to BI (f^2^ = 1.208), PP to both PU (f^2^ = 1.027) and PEOU (f^2^ = 0.687), and PI to PU (f^2^ = 0.416). PI has a medium effect on PEOU (f^2^ = 0.270), while PEOU has a small effect on BI (f^2^ = 0.040) and PU (f^2^ = 0.102). All of the significant paths of the proposed model show at least a weak effect size greater than 0.020, while the majority of the four out of a total of seven paths show large effect sizes, much greater than 0.350.

### 4.2. Findings and Discussion

This study contributes to the acceptance of AR technology by firefighters, by combining the variables PU, PEOU, and BI from the core TAM model [31], and two more external variables, namely, PI and PP. Our findings have implications for academia, as the acceptance of information systems technology by firefighters is an under-researched area. The results of our study provide a good explanation of the domain-specific acceptance model.

Regarding the results of the outer model, the indicators’ reliability and the internal consistency are confirmed. Indeed, all the factor loadings ranged from 0.833 to 0.992, which are greater than the 0.700 threshold limit. As far as the internal consistency of the variables is concerned, the CA values ranged from 0.902 to 0.981, while the CR ranged from 0.940 to 0.986. Sagnier et al. [42] chose to use CR rather than CA for internal consistency, justifying their approach in the conservative measurement of CA. However, we assessed the quality of the constructs by using both CA and CR, which were greater than the 0.700 cut-off value. To examine the convergent validity, we assessed the AVE value of each construct, which ranged from 0.839 to 0.959, exceeding 0.500.

Furthermore, the model’s discriminant validity is assessed by two means, first by the AVE square root of each variable, and secondly by the HTMT ratio of correlations. Pal and Patra [41] also used both means, based on the criticism about the use of the Fornell and Larcker [55] criterion for measuring the discriminant validity as not always an effective mean for evaluating this type of validity. Our measurements of the AVE square root values of the inter-item correlation matrix supported the conditions of discriminant validity. With respect to the HTMT values, the correlation between the constructs ranged from 0.809 to 0.882, less than the recommended threshold value of 0.950, confirming the existence of discriminant validity alternatively.

Regarding the results of the inner model, the findings showed that the BI target endogenous variable has a coefficient of determination (R^2^) value of 0.910, meaning that PU and PEOU explain 91.0% of the variance of BI. Moreover, PI and PP explain 84.0% of PEOU, while PI, PP, and PEOU explain 94.6% of the variance of PU.

The structural model suggests that PU has the strongest effect on BI (0.822), while PEOU has a weaker effect on BI (0.142). Both hypothesized path relationships between PU and BI, and PEOU and BI, are statistically significant as their t-statistics values, 17.790 and 2.876, respectively, are greater than the cut-off value of 2.580. Furthermore, PEOU is highly affected firstly by PP (0.593) and secondly by PI (0.363). As far as PU is concerned, it is affected firstly by PP (0.542), secondly by PI (0.294), but also from PEOU (0.182).

From the evaluation results presented in this section, it can be observed that the behavioral intention to use the AR application is highly affected by usefulness, while ease of use has a much weaker effect. In our study, we found a positive, yet comparably smaller, effect of the perceived ease of use on user acceptance of AR, either directly on the behavioral intention to use, or indirectly through the perceived usefulness. It seems reasonable considering the domain of firefighting, which involves critical situations with people’s lives at stake. Firefighters demand an AR application for their training, firstly absolutely useful for real-life scenarios, and secondly to be easy to use. They seem willing to tolerate a certain amount of difficulty to handle a technology application, as far as the usability is proper. These findings are in line with previous studies [20,64], which consider perceived usefulness to be a strong predictor of intention to use.

The perceived usefulness is also very well predicted by the external variables of interactivity and personalization, which were included in our proposed model in order to evaluate the system and the individual level, respectively. As far as the perceived interactivity is concerned, we found significance in the role of the communication, the user control, and the time. By implication, it suggests that firefighters (a) appreciate two-way communication through the AR application, (b) enjoy the flexibility of controlling a mobile device for their training, thus operating virtual objects, and (c) need fast-delivered messages and information, as their reactions have to be immediate and effective.

As far as the perceived personalization is concerned, we also found significance for the application’s usability. Indeed, firefighting is a process where numerous operations occur simultaneously. As such, firefighters can participate in fire service training through various training formats, encouraging personal and professional growth. The AR firefighting application offers guidance and support, in order to assist personally every trainee to navigate through the competitive process of attaining a career as a firefighter. The participants experience one-on-one personal coaching through virtual classroom training sessions.

The perceived interactivity positively affects not only the system’s usability, but also the perceived ease of use. Indeed, in the fire service, the hands-on portion of the training is based on the interactivity of the training platform. The must-have features, as well as the right support to assist with the AR functionality, give credits to the trainees’ ease to use the AR application, increasing the retention and the actual system usage.

The perceived personalization equally affected usefulness and ease of use. A training system tailored to the firefighters’ needs provides the personnel self-paced training, with greater consistency. The better the AR application fits to the firefighters needs, the easier and more convenient the training is. On the contrary, traditional in-person training sessions are typically one-size-fits-all. The trainees attend the same training session, regardless of their roles, i.e., ship crew or officers.

In summary, our findings provide three meaningful implications for the user acceptance of AR firefighting training. The first one is that the external variable of perceived interactivity has a positive impact on the perceived usefulness and perceived ease of use. The second one is that the second external variable of perceived personalization has also a positive, but stronger than interactivity, impact on the perceived usefulness and perceived ease of use. The third one is that usability is the strongest predictor of behavioral intention; however, the direct and indirect effect of ease of use is still meaningful. The perception of interaction experienced by firefighters is about controlling the content, the communication, and the speed, and the personalization on the feedback of their actions. These factors influence the intrinsic motivation of the firefighters, their perception of the ease of use of the AR application, and the perceived usefulness. The contribution in the field of AR, based on the study of the proposed TAM model, is that the use of AR technology may be prevalent in firefighting training, and may become an integral part of the educational procedure. This assumption is justified by the fact that firefighting is a high-risk job and firefighters are exposed to danger, so they prioritize their safety. The evaluation results show that AR can potentially become a useful technology upgrade for an immersive training, so that firefighters would be prepared for high-risk incidents.

In our proposed model, usability was found to be the strongest predictor of the intention to use AR in firefighting training. The variable of usability is crucial and, as such, the results can be expanded in other domains such as aviation and military. Indeed, the aforementioned domains base their training on highly usable applications, combining both interactive and adaptive techniques.

## 5. Conclusions and Future Work

The presented technology acceptance model contributes to achieving an in-depth understanding as to why firefighters accept or reject an AR firefighting training application (i.e., NAFTES). The training advantages of cost-effectiveness, variety of real-life scenarios, and safety, offered by the AR platform, made us consider a novel, well-established model to measure the user experience and usability of the training application. The results of the conducted evaluation additionally provide insights into the significance and the comparative importance of the external variables, thus providing better support for their ongoing development.

Regarding the limitations and the future steps of this research, the study involved trainees from Greece, who possibly exhibited some common features in terms of user experience. Therefore, it is possible that our findings may possess limited generalization, so future research should involve more countries, aiming at enhancing the quality of the training of ship crews in firefighting operations.

## Figures and Tables

**Table 1:** Demographics.

Measure	Item	Frequency	Percentage (%)
Sample size		200	100.0
Gender	Male	136	68.0
	Female	64	32.0
Age (18–60)	Below 22	48	24.0
	23–34	72	36.0
	35–49	57	28.5
	Over 50	23	11.5
Professional qualification	Under 1 year	128	64.0
	1–5 years	51	25.5
	Over 5 years	21	10.5

**Table 2:** Questionnaire details used in the survey.

Constructs	Indicator	Questionnaire	Source
Perceived interactivity (PI)	PI1	I felt that I had a lot of control over my experiences of using the AR application.	Yoo et al. [44]
	PI2	Getting information from the AR application was very fast.	
	PI3	I think using the AR application was enjoyable.	Zhao and Lu [45]
Perceived personalization (PP)	PP1	I value AR application that is personalized for the device that I use.	Hubert et al. [39]
	PP2	I value AR application that is personalized for my usage experience preference.	
	PP3	I value AR application that acquires my personal preferences and personalizes the services themselves.	
Perceived usefulness (PU)	PU1	Using AR application improves my learning performance.	Davis [20]
	PU2	Using AR application makes my training more productive.	
	PU3	Using AR application enhances my effectiveness on my training.	
	PU4	Overall, I find AR application useful in my job.	
Perceived ease of use (PEOU)	PEOU1	Learning to operate AR application is easy for me.	Davis [20]
	PEOU2	I find it easy to get AR application to do what I want to do.	
	PEOU3	My interaction with the AR application is clear and understandable.	
	PEOU4	Overall, I find AR application easy to use.	
Behavioral intention to use (BI)	BI1	Using AR application enhances my training interest.	Fishbein and Ajzen [30]
	BI2	I intend to use AR application for training in the future.	
	BI3	I will recommend others to use AR application for training.	

**Table 3:** Descriptive statistics of the indicators.

Indicator	M	SD	Kurtosis	Skewness
BI1	5.630	1.050	−0.273	−0.543
BI2	5.540	1.117	−0.349	−0.491
BI3	5.470	1.131	−0.332	−0.583
PEOU1	5.125	1.208	−0.494	−0.277
PEOU2	5.455	1.191	−0.204	−0.528
PEOU3	4.955	1.242	−0.618	−0.088
PEOU4	5.265	1.321	−0.094	−0.499
PI1	5.030	1.067	−0.631	−0.010
PI2	6.160	0.771	0.935	−0.908
PI3	5.210	1.130	−0.734	−0.191
PP1	5.210	1.130	−0.734	−0.191
PP2	5.380	1.164	−0.676	−0.396
PP3	4.940	1.240	−0.915	0.083
PU1	5.240	1.040	−0.737	0.042
PU2	5.200	1.058	−0.484	−0.026
PU3	5.640	1.044	−0.217	−0.563
PU4	5.440	1.160	−0.719	−0.298

**Table 4:** Measurement model’s reliability and convergent validity.

Construct	Indicator	Outer Loading	CA	CR	AVE
BI	BI1	0.992	0.979	0.986	0.959
	BI2	0.978			
	BI3	0.969			
PEOU	PEOU1	0.970	0.981	0.986	0.947
	PEOU2	0.975			
	PEOU3	0.961			
	PEOU4	0.985			
PI	PI1	0.949	0.902	0.940	0.839
	PI2	0.833			
	PI3	0.961			
PP	PP1	0.983	0.976	0.985	0.955
	PP2	0.977			
	PP3	0.972			
PU	PU1	0.933	0.967	0.976	0.911
	PU2	0.947			
	PU3	0.971			
	PU4	0.966			

**Table 5:** Measurement model’s discriminant validity (Fornell Larcker criterion).

	BI	PEOU	PI	PP	PU
BI	0.979				
PEOU	0.896	0.973			
PI	0.900	0.854	0.916		
PP	0.909	0.894	0.828	00.977	
PU	0.952	0.918	0.899	00.949	00.954

**Table 6:** HTMT results.

	BI	PEOU	PI	PP	PU
BI					
PEOU	0.814				
PI	0.859	0.809			
PP	0.830	0.813	0.882		
PU	0.876	0.841	0.861	0.876	

**Table 7:** Structural model’s results.

Hypothesis	Path	β Coefficients	t-Statistics	*p*-Value	Supported or Not
H1	PEOU → PU	0.182	40.610	0.000	Yes
H2	PEOU → BI	0.142	20.876	0.004	Yes
H3	PU → BI	0.822	170.790	0.000	Yes
H4	PI → PU	0.294	100.417	0.000	Yes
H5	PI → PEOU	0.363	60.988	0.000	Yes
H6	PP → PU	0.542	190.631	0.000	Yes
H7	PP → PEOU	0.593	110.891	0.000	Yes

**Table 8:** R^2^ and Q^2^ results.

Constructs	R^2^	Q^2^
BI	0.910	0.867
PEOU	0.840	0.790
PU	0.946	0.855

**Table 9:** f^2^ results.

	BI	PEOU	PU
BI			
PEOU	0.040		0.102
PI		0.270	0.416
PP		0.687	1.027
PU	1.208		

## Data Availability

Data available on request.
